# Utilization and spending trends for antiretroviral medications in the U.S. Medicaid program from 1991 to 2005

**DOI:** 10.1186/1742-6405-4-22

**Published:** 2007-10-16

**Authors:** Yonghua Jing, Patricia Klein, Christina ML Kelton, Xing Li, Jeff J Guo

**Affiliations:** 1 College of Pharmacy, University of Cincinnati Medical Center, Cincinnati, Ohio, USA; 2 College of Business, University of Cincinnati, Cincinnati, Ohio, USA; 3 Institute for the Study of Health, University of Cincinnati, Cincinnati, Ohio, USA; 4 Professor of Pharmacoeconomics & Pharmacoepidemiology, Division of Pharmacy Practice and Administrative Sciences, University of Cincinnati College of Pharmacy, 3225 Eden Ave., Cincinnati, OH 45267-0004, USA

## Abstract

**Background:**

HIV/AIDS incidence and mortality rates have decreased in the U.S. since 1996. Accompanying the longer life spans of those diagnosed with the disease, however, is a tremendous rise in expenditures on medication. The objective of this study is to describe the trends in utilization of, spending on, and market shares of antiretroviral medications in the U.S. Medicaid Program. Antiretroviral drugs include nucleoside reverse transcriptase inhibitors (NRTIs), protease inhibitors (PIs), nonnucleoside reverse transcriptase inhibitors (NNRTIs), and fusion inhibitors (FIs).

**Methods:**

Utilization and payment data from 1991 to 2005 are provided by the Centers for Medicare & Medicaid Services. Descriptive summary analyses were used to assess quarterly prescription numbers and amounts of payment.

**Results:**

The total number of prescriptions for antiretrovirals increased from 168,914 in 1991 to 2.0 million in 1998, and 3.0 million in 2005, a 16.7-fold increase over 15 years. The number of prescriptions for NRTIs reached 1.6 million in 2005. Prescriptions for PIs increased from 114 in 1995 to 932,176 in 2005, while the number of prescriptions for NNRTIs increased from 1,339 in 1996 to 401,272 in 2005. The total payment for antiretroviral drugs in the U.S. Medicaid Program increased from US$ 30.6 million in 1991 to US$ 1.6 billion in 2005, a 49.8-fold increase. In 2005, NRTIs as a class had the highest payment market share. These drugs alone accounted for US$ 787.9 million in Medicaid spending (50.8 percent of spending on antiretrovirals). Payment per prescription for each drug, with the exception of Agenerase^®^, increased, at least somewhat, over time. The relatively expensive drugs in 2005 included Trizivir^® ^($1040) and Combivir^® ^($640), as well as Reyataz^® ^($750), Lexiva^® ^($700), Sustiva^® ^($420), Viramune^® ^($370), and Fuzeon^® ^($1914).

**Conclusion:**

The tremendous growth in antiretroviral spending is due primarily to rising utilization, secondarily to the entry of newer, more expensive antiretrovirals, and, finally, in part to rising per-prescription cost of existing medications.

## Background

Since the first reported case in June 1981, approximately 1.7 million people in the United States have been infected with HIV, including more than 550,000 who have already died and an estimated 1.2 million living with HIV/AIDS in 2005 [1-4]. AIDS cases increased rapidly in the 1980s and peaked in 1992 (an estimated 78,000 cases diagnosed) before stabilizing in 1998; since then, approximately 40,000 AIDS cases have been diagnosed annually, although, over the last several years, there is some indication that diagnoses are again on the rise [5]. The HIV/AIDS-related mortality rate rose steadily through the 1980s, peaking in 1994–1995 [6] and declining since then.

There is no known cure for AIDS. Patients infected with HIV rely on antiretroviral treatment, a life-long disease management strategy, costing between US$10,000 and US$15,000 per year [7]. Once drugs from several antiretroviral drug classes were available beginning in 1996, the introduction of therapies incorporating combinations of drugs from two or three different drug classes has led to a wide range of possible antiretroviral therapy combinations. These new combinations, often referred to as highly active antiretroviral therapy (HAART), have been shown to have a significant impact both on markers of disease progression (viral load and CD4 T-cell counts) [8-10] and on HIV/AIDS-associated mortality and morbidity [6,11-14]. National guidelines for the treatment of HIV infection recommend HAART as first-choice therapy [15].

Accompanying HAART and the longer survival rates offered by HAART has been a marked rise in expenditures on antiretroviral medications. The U.S. Medicaid Program bears a substantial burden in HIV/AIDS expense coverage, especially in the latter stages of the disease when individuals are too ill to work. The objective of this study is to describe the trends of utilization of and spending on antiretroviral drug classes as well as individual antiretroviral medications in the U.S. Medicaid Program. These results provide useful information to policy makers and health professionals interested in cost-effectiveness and cost-containment strategies along with their usual concerns of safety and efficacy.

## Methods

We study utilization, spending, and market share for each of the antiretrovirals listed in Table 1[16]. Each drug is classified as a nucleoside reverse transcriptase inhibitor (NRTI), a protease inhibitors (PI), a nonnucleoside reverse transcriptase inhibitor (NNRTI), or a fusion inhibitor (FI). Its manufacturer, approval date, and time to approval (after new drug application submission) are all identified. Note the relatively short pre-approval periods for all of the drugs due to the AA priority status for AIDS therapy applications [17]. (As of 2003, the average time to approval for all drugs was around one and a half years [18].)

**Table 1:** Antiretroviral Medications Purchased by the U.S. Medicaid Program from 1991 to 2005

**Brand Name**	**Generic Name(s)**	**Manufacturer**	**Approval Date**	**Time to Approval**
**Nucleoside Reverse Transcriptase Inhibitors (NRTIs)**				
Combivir	lamivudine and zidovudine	GlaxoSmithKline	27-Sep-97	3.9 months
Emtriva	emtricitabine, FTC	Gilead Sciences	02-Jul-03	10 months
Epivir	lamivudine, 3TC	GlaxoSmithKline	17-Nov-95	4.4 months
Epzicom	abacavir and lamivudine	GlaxoSmithKline	02-Aug-04	10 months
Hivid	zalcitabine, dideoxycytidine, ddC	Hoffmann-La Roche	19-Jun-92	7.6 months
Retrovir	zidovudine, azidothymidine, AZT, ZDV	GlaxoSmithKline	19-Mar-87	3.5 months
Trizivir	abacavir, zidovudine, and lamivudine	GlaxoSmithKline	14-Nov-00	10.9 months
Truvada	tenofovir disoproxil fumarate and emtricitabine	Gilead Sciences, Inc.	02-Aug-04	5 months
Videx EC	enteric coated didanosine, ddI EC	Bristol Myers-Squibb	31-Oct-00	9 months
Videx	didanosine, dideoxyinosine, ddI	Bristol Myers-Squibb	9-Oct-91	6 months
Viread	tenofovir disoproxil fumarate, TDF	Gilead	26-Oct-01	5.9 months
Zerit	stavudine, d4T	Bristol Myers-Squibb	24-Jun-94	5.9 months
Ziagen	abacavir sulfate, ABC	GlaxoSmithKline	17-Dec-98	5.8 months
**Protease Inhibitors (PIs)**				
Agenerase	amprenavir, APV	GlaxoSmithKline	15-Apr-99	6 months
Aptivus	tipranavir, TPV	Boehringer Ingelheim	22-Jun-05	6 months
Crixivan	indinavir, IDV	Merck	13-Mar-96	1.4 months
Fortovase	saquinavir (no longer marketed)	Hoffmann-La Roche	7-Nov-97	5.9 months
Invirase	saquinavir mesylate, SQV	Hoffmann-La Roche	6-Dec-95	3.2 months
Kaletra	lopinavir and ritonavir, LPV/RTV	Abbott Laboratories	15-Sep-00	3.5 months
Lexiva	Fosamprenavir Calcium, FOS-APV	GlaxoSmithKline	20-Oct-03	10 months
Norvir	ritonavir, RTV	Abbott Laboratories	1-Mar-96	2.3 months
Reyataz	atazanavir sulfate, ATV	Bristol-Myers Squibb	20-Jun-03	6 months
Viracept	nelfinavir mesylate, NFV	Agouron Pharmaceuticals	14-Mar-97	2.6 months
**Nonnucleoside Reverse Transcriptase Inhibitors (NNRTIs)**				
Rescriptor	delavirdine, DLV	Pfizer	4-Apr-97	8.7 months
Sustiva	efavirenz, EFV	Bristol Myers-Squibb	17-Sep-98	3.2 months
Viramune	nevirapine, NVP	Boehringer Ingelheim	21-Jun-96	3.9 months
**Fusion Inhibitors**				
Fuzeon	enfuvirtide, T-20	Hoffmann-La Roche & Trimeris	13-Mar-03	6 months

Source: Food And Drug Administration: [16]

Retrospective descriptive summary analyses were conducted with the purpose of describing the trends in utilization of and spending on antiretroviral medications over the last 15 years. Pharmacy utilization and expenditure data, from 1991 quarter 1 through 2005 quarter 4, were taken from the national Medicaid pharmacy files provided by the Centers for Medicare & Medicaid Services (CMS). These files contain number of outpatient prescriptions and payment amounts for all National Drug Code (NDC) drug forms paid for by any state (except Arizona, but including the District of Columbia) Medicaid program [19]. The national files are huge databases representing aggregation across all the states and are subject to occasional coding error, which we corrected to the best of our ability. Prescription and expenditure data were then aggregated across all NDCs for each of the drugs listed in Table 1. Per-prescription spending (which is referred to loosely as "price" throughout this article) was calculated as total expenditure for the drug divided by total number of prescriptions. Note that per-prescription spending exceeds actual acquisition cost to Medicaid due to federal and state rebates received by Medicaid from the drug manufacturers.

Quarterly market shares for four classes of antiretroviral medications are calculated as both the percentage of total antiretroviral prescription numbers and the percentage of total antiretroviral expenditures in the U.S. Medicaid market. We refer to these market shares as the prescription market share and the payment market share, respectively. All expenditure values are expressed in current U.S. dollars.

## Results

**Figure 1:**
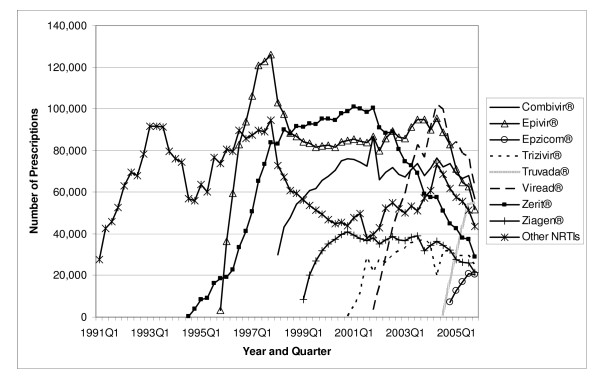
**Utilization of NRTI Antiretrovirals by Quarter in Medicaid: 1991–2005**. Note: Other NRTIs include didanosine, Emtriva^®^, Hivid^®^, Retrovir^®^, and Videx^®^.

**Figure 2:**
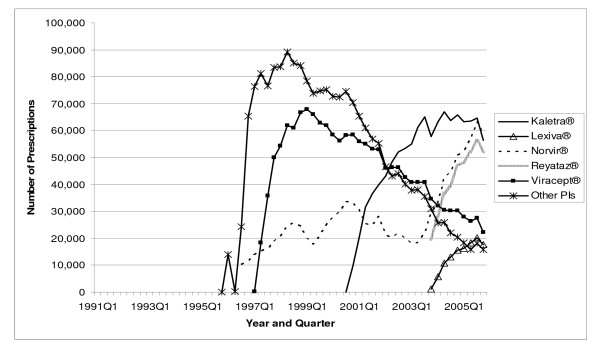
**Utilization of PI Antiretrovirals by Quarter in Medicaid: 1991–2005**. Note: Other PIs include Agenerase^®^, Aptivus^®^, Crixivan^®^, and Invirase/Fortovase^®^.

**Figure 3:**
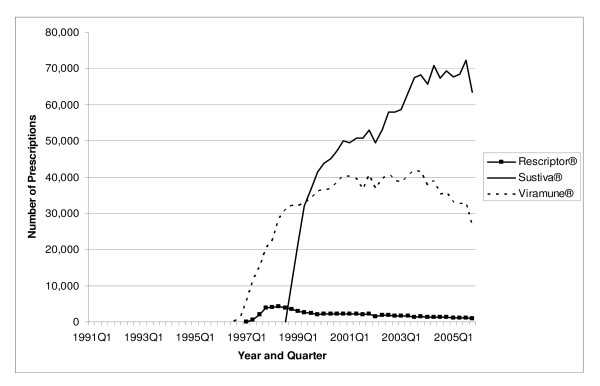
Utilization of NNRTI Antiretrovirals by Quarter in Medicaid: 1991–2005.

The total number of antiretroviral prescriptions paid for by Medicaid increased from 168,914 in 1991 to 3.0 million in 2005, showing a 16.7-fold increase over 15 years (see Table 2). In 1996, and again in 1997, utilization of antiretrovirals increased by more than 100 percent in a single year. The number of prescriptions for NRTIs increased from 168,914 in 1991 to 1.6 million in 2005. Prescriptions for PIs increased from 114 in 1995 to 932,176 in 2005. The number of prescriptions for NNRTIs increased from 1,339 in 1996 to 401,272 in 2005. The prescriptions for the most recent class of antiretrovirals, the FIs, increased from 6,683 in 2003 to 20,391 in 2005, representing a 205.1 percent increase in just two years. As shown in Figure 1, in 2005 quarter 4, the three NRTI market leaders were Viread^® ^with 62,513 prescriptions, Combivir^® ^with 56,735 prescriptions, and Truvada^® ^with 54,788 prescriptions. Included in "Other NRTIs" (each with no more than 17,000 prescriptions in 2005 quarter 4) are the two original NRTIs, Retrovir^® ^and Videx^®^, which still play some role in the Medicaid market. In 2005 quarter 4, Medicaid prescriptions for Retrovir^® ^totaled 9,970, while those for Videx^® ^plus didanosine totaled 28,213. Figure 2 shows the three PI market leaders. In 2005 quarter 4, there were 58,605 prescriptions for Norvir^®^, 56,327 prescriptions for Kaletra^®^, and 51,973 prescriptions for Reyataz^®^. Most of the "Other PIs" (again, each with no more than 17,000 prescriptions in 2005 quarter 4), with the exception of the newest entrant Aptivus^®^, have experienced a declining number of prescriptions over time, as can be seen in Figure 2. Finally, Figure 3 identifies Sustiva^® ^as the NNRTI market leader in 2005.

**Table 2:** Annual Medicaid Prescriptions and Prescription Market Shares for Antiretroviral Medications: 1991 – 2005

**Year**	**Number of Prescriptions**						**Prescription Market Share**			
	
	**Annual Rx**	**Percent Annual Increase**	**NNRTI**	**NRTI**	**PI**	**FI**	**NNRTI**	**NRTI**	**PI**	**FI**
1991	168,914			168,914				100.0		
1992	278,873	65.1		278,873				100.0		
1993	354,082	27.0		354,082				100.0		
1994	267,411	-24.5		267,411				100.0		
1995	329,843	23.3		329,729	114			100.0		
1996	792,949	140.4	1,339	665,261	126,349		0.2	83.9	15.9	
1997	1,656,122	108.9	59,742	1,109,954	486,426		3.6	67.0	29.4	
1998	1,985,866	19.9	140,839	1,162,838	682,189		7.1	58.6	34.4	
1999	2,167,620	9.2	277,094	1,246,271	644,255		12.8	57.5	29.7	
2000	2,345,000	8.2	346,993	1,342,463	655,544		14.8	57.2	28.0	
2001	2,510,715	7.1	369,722	1,447,701	693,292		14.7	57.7	27.6	
2002	2,588,815	3.1	381,667	1,571,995	635,153		14.7	60.7	24.5	
2003	2,793,043	7.9	425,868	1,713,116	647,376	6,683	15.2	61.3	23.2	0.2
2004	3,068,032	9.8	426,253	1,780,188	844,116	17,475	13.9	58.0	27.5	0.6
2005	2,988,138	-2.6	401,272	1,634,299	932,176	20,391	13.4	54.7	31.2	0.7

Source: Centers for Medicare & Medicaid Services: **State drug utilization data**. [27].

**Figure 4:**
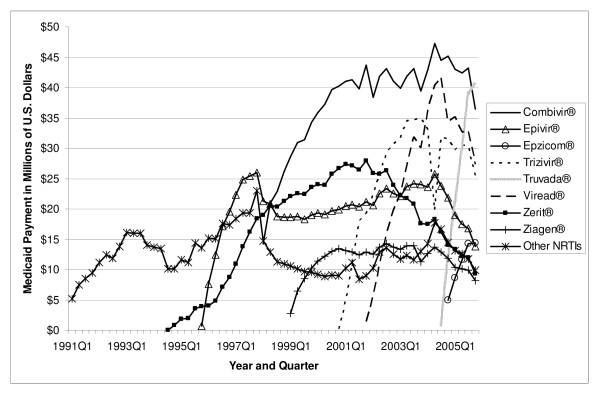
**Payment for NRTI Antiretrovirals by Quarter in Medicaid: 1991–2005**. Note: Other NRTIs include didanosine, Emtriva^®^, Hivid^®^, Retrovir^®^, and Videx^®^.

**Figure 5:**
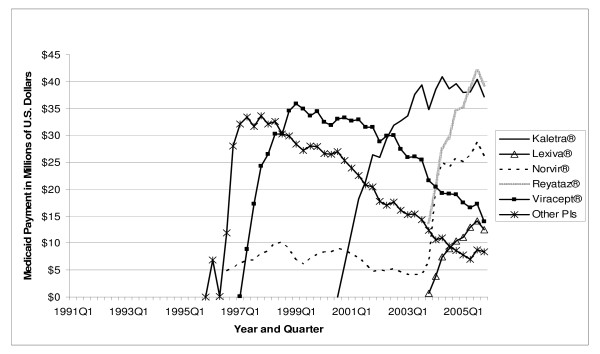
**Payment for PI Antiretrovirals by Quarter inMedicaid: 1991–2005**. Note: Other PIs include Agenerase^®^, Aptivus^®^, Crixivan^®^, and Invirase/Fortovase^®^.

**Figure 6:**
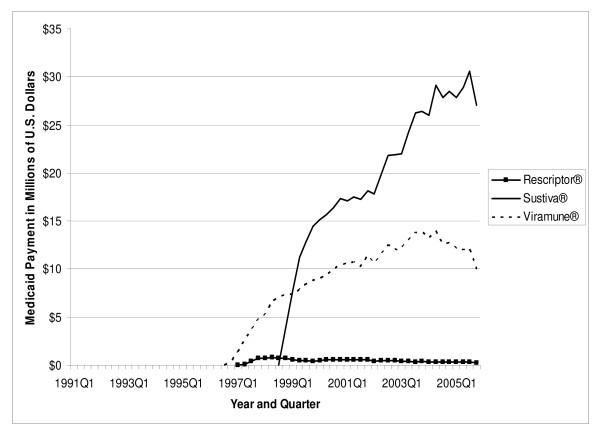
Payment for NNRTI Antiretrovirals by Quarter in Medicaid: 1991–2005.

Total U.S. Medicaid expenditure on antiretroviral medications increased from US$ 30.6 million in 1991 to US$ 1.6 billion in 2005, a 49.8-fold increase (Table 3). In 2005, NRTIs as a class had the highest payment market share, accounting for US$ 787.9 million in Medicaid spending (50.8 percent of total spending on antiretrovirals). PIs came in second at US$ 563.5 million (36.3 percent), and NNRTIs were third at US$ 161.6 million (10.4 percent of spending). As of 2005, the FI class accounted for only a 2.5 percent payment market share. Figures 4, 5, and 6 show the trend of payments for each drug in the NRTI, PI, and NNRTI classes, respectively; these figures tell a similar story to that of Figures 1, 2, and 3. The three most costly NRTIs in 2005 quarter 4 were Truvada^®^, Combivir^®^, and Viread^® ^(Figure 4). When the first generic antiretroviral, didanosine (for Videx^®^), became available in 2004, the spending on Videx^® ^decreased from US$ 8.6 million in the fourth quarter of 2004 to US$ 2.6 million in the fourth quarter of 2005. (Note that Videx^® ^is not shown individually in Figure 4; it is part of the category "Other NRTIs.") Meanwhile, the spending on generic didanosine (also among the "Other NRTIs") increased from US$ 15,207 in the fourth quarter of 2004 to US$ 3.2 million in the fourth quarter of 2005. The three most costly PIs in 2005 were Reyataz^®^, Kaletra^®^, and Norvir^® ^(Figure 5), while Sustiva^® ^was the market leader in the NNRTI class. The spending on Sustiva^® ^increased from US$ 3,431 in the third quarter of 1998 to US$ 27.0 million in the fourth quarter of 2005 (Figure 6). In 2005, Sustiva^® ^accounted for US$ 114.3 million in Medicaid spending (70.7 percent of spending on NNRTIs) over four quarters.

**Table 3:** Annual Medicaid Payments and Payment Market Shares for Antiretroviral Medications: 1991 – 2005

**Year**	**Payment Amounts**						**Payment Market Share**			
	
	**Annual Amount ($)**	**Percent Annual Increase**	**NNRTI**	**NRTI**	**PI**	**FI**	**NNRTI**	**NRTI**	**PI**	**FI**
1991	30,581,688			30,581,688				100.0		
1992	49,145,345	60.7		49,145,345				100.0		
1993	62,163,949	26.5		62,163,949				100.0		
1994	48,314,392	-22.3		48,314,392				100.0		
1995	62,667,235	29.7		62,613,187	54,047			99.9	0.1	
1996	192,066,249	206.5	321,552	134,207,229	57,537,468		0.2	69.9	30.0	
1997	460,706,553	139.9	13,537,612	237,889,361	209,279,580		2.9	51.6	45.4	
1998	612,308,495	32.9	33,374,299	294,685,007	284,249,188		5.5	48.1	46.4	
1999	717,516,585	17.2	80,748,122	358,063,912	278,704,550		11.3	49.9	38.8	
2000	799,546,390	11.4	105,358,639	417,270,377	276,917,374		13.2	52.2	34.6	
2001	936,652,425	17.1	115,254,370	501,229,292	320,168,762		12.3	53.5	34.2	
2002	1,073,255,281	14.6	129,858,945	618,622,608	324,773,728		12.1	57.6	30.3	
2003	1,190,098,926	10.9	153,303,569	689,086,945	335,785,345	11,923,067	12.9	57.9	28.2	1.0
2004	1,452,823,690	22.1	165,523,923	741,094,660	513,999,395	32,205,713	11.4	51.0	35.4	2.2
2005	1,552,004,168	6.8	161,640,662	787,864,253	563,473,942	39,025,310	10.4	50.8	36.3	2.5

Source: Centers for Medicare & Medicaid Services: **State drug utilization data**. [27].

The average payment per prescription for NRTIs increased from US$ 181 in 1991 to US$ 482 in 2005 (Table 4). The average per-prescription payment for PIs increased from US$ 474 in 1995 to US$ 605 in 2005. The average for NNRTIs increased from US$ 240 in 1996 to US$ 403 in 2005. The newest antiretroviral medication, Fuzeon^®^, entered the Medicaid market with a very high price (US$ 1784 in 2003); the price increased to US$ 1914 by 2005. Spending per prescription for individual drugs can be calculated straightforwardly by dividing spending (Figure 4, 5, or 6) by number of prescriptions (Figure 1, 2, or 3). At the beginning of the study period, a single NRTI (Retrovir^®^) had payment per prescription of US$ 180 (slightly below the US$ 200 price point). As newer NRTIs entered the market, they did so around the US$ 200 level. However, four of the more recent NRTIs entered at higher price points (Viread^® ^and Ziagen^® ^at around the US$ 400 level, Combivir^® ^at approximately the US$ 600 level, and Trizivir^® ^at around the US$ 900 level). The two most recent entries, Truvada^® ^in 2004 quarter 3 and Epzicom^® ^in 2004 quarter 4, entered at US$ 714 and US$ 674, respectively. Once an NRTI enters the market, its per-prescription payment is seen to rise over time, though not that rapidly. For example, since Trizivir's^® ^entry in 2000 quarter 4, its price has risen 21.4 percent (as of 2005 quarter 4). Over the same five years, the price of Retrovir^® ^has risen 10.5 percent. In some ways, the prices in the PI class have behaved similarly to those of the NRTIs. We see, though with more variability over time, the upward trend in individual prices, except for Viracept ^® ^(high initial price, followed by a considerable price drop, followed by a gradual increase over time), and Norvir^® ^(gradual decrease in price, followed by an abrupt price increase in 2004 quarter 1 and then followed by again a gradual decline). In 2005 quarter 4, prices for a drug in the PI class ranged from a little less than US$ 300 (Agenerase^®^) to US$ 926 (Aptivus^®^, the latest entry to the PI class in 2005 quarter 3). (Neither of these drugs is featured individually but contributes to the category "Other PIs.") Similarly to the NRTIS, the later PI entrants (such as Lexiva^®^, Kaletra^®^, and Reyataz^®^, and certainly Aptivus^®^) have entered at higher price points than their earlier counterparts. Finally, the data tell a similar story for the NNRTIs. Sustiva^®^, market leader and latest entrant to the market, entered at a higher price than either Rescriptor^® ^(ignoring its price in the first quarter for Medicaid) or Viramune^®^. Each of the three drugs has shown a price increase over time. Since 1998 quarter 3, Rescriptor^® ^has had a price increase of 37.3 percent, Sustiva^® ^of 24.2 percent, and Viramune^® ^of 62.0 percent, as of 2005 quarter 4.

**Table 4:** Annual Medicaid Payment per Prescription for Antiretroviral Medications: 1991 – 2005

**Year**	**Average Per-Rx $ Payment**			
	
	**NNRTI**	**NRTI**	**PI**	**FI**
1991		181.0		
1992		176.2		
1993		175.6		
1994		180.7		
1995		189.9	474.1	
1996	240.1	201.7	455.4	
1997	226.6	214.3	430.2	
1998	237.0	253.4	416.7	
1999	291.4	287.3	432.6	
2000	303.6	310.8	422.4	
2001	311.7	346.2	461.8	
2002	340.2	393.5	511.3	
2003	360.0	402.2	518.7	1784.1
2004	388.3	416.3	608.9	1843.0
2005	402.8	482.1	604.5	1913.8

Source: Centers for Medicare & Medicaid Services: **State drug utilization data**. [27]

## Discussion

In the preceding section, we showed a substantial rise in Medicaid expenditures on antiretroviral medications. While most of that rise can be explained by the rise in utilization of antiretroviral medicines (this utilization increase is observed in the face of decreasing or stable HIV/AIDS incidence rates in the U.S.), some is certainly attributable to both the entry of newer, more expensive drugs in all of the antiretroviral drug classes and price increases of drugs once they are being marketed.

The first factor driving the increase in Medicaid prescriptions over the past decade and a half is the move from monotherapy to combination therapy. In a short period of time after the approval of the PIs, HAART became the standard treatment for those infected with HIV, implying an increase in the use of double- and, more recently, triple-combination antiretroviral therapy regimens, with drugs across antiretroviral drug classes, among HIV-infected persons. Medicaid patients consistently, however, have lower use rates for the newer antiretroviral drugs than the general population [20,21]; estimates for various states indicate that HAART use among HIV-positive patients on Medicaid in 1998 ranged from 37 percent in Texas to almost 70 percent in New Jersey [22,23].

Secondly, rising utilization can be explained by declining mortality rates, leading to individuals' requiring more prescriptions over life's course. Using data from a random-assignment clinical trial, it was found that those assigned HAART therapy had a 58 percent lower mortality rate than those in the control group [24]. Survival data for patients in non-experimental settings have demonstrated that patients using HAART therapy have substantially lower mortality rates than those not using it.

During the study period, more than twenty marketed antiretrovirals were supplied by nine manufacturers. The data indicate that new entry occurs over time at higher and higher price points. Price differences among the antiretrovirals are explained to some degree by differences in effectiveness and safety profiles. Newer drugs that offer added dosing convenience and improved safety profiles are priced higher than previously popular drugs that are being used less often as their drawbacks become better defined and drug resistance is developed [15]. The data indicate as well that payment per prescription has risen over time for existing drugs in the market. The rising demand generated by both combination therapy and higher survival rates has overwhelmed any downward pressures on price.

Once a drug goes off patent, generic versions of the drug quickly enter the market. The number of entrants depends on the size of the market. A large number of generic producers will ideally drive price down to a level that is close to marginal cost. However, while didanosine (since 2004 quarter 4) and zidovudine (since 2005 quarter 4) are now available as generic drugs, low-cost versions of these two NRTIs have not yet made a big impact on the expenditure on antiretroviral medication for Medicaid. At this point, there are very few generic manufacturers. As of 2005 quarter 4, only *one *generic producer supplied didanosine to Medicaid. In 2005 quarter 4, the number of prescriptions for Videx^® ^(including Videx EC^®^) was still 12,128 (payment per prescription was $218). Comparatively, the number of prescriptions for the generic form of Videx^®^, didanosine, was 16,085; spending per prescription was $201, not much lower than the brand-name price. Four generic producers sold zidovudine in 2005 quarter 4. That quarter, the average price of zidovudine, across the four manufacturers and all their NDCs, was $211. Compared to the price of Retrovir^®^, $248, the generic producers were selling their drug for 85 percent of the brand-name price.

### Study limitations

Drug use was inferred from aggregate prescription data, themselves based on adding up prescriptions and payments from Medicaid claims data; it was not possible to review patient-specific clinical information. We were unable to analyze the use of antiretrovirals as a function of disease treated, and unable to link the utilization with patient demographics or history of clinical conditions and comorbidities. We did not link the utilization of antiretrovirals to the prevalence rate of HIV or AIDS.

### Policy implications

Medicaid plays a critical role in financing AIDS care by providing a comprehensive benefit package that includes prescription drugs [25]. The rising cost of antiretroviral medications has been a significant challenge for all state Medicaid programs in the United States. Because the antiretrovirals are protected by patents, with the recent exceptions of didanosine and zidovudine, aggressive generic substitution policies cannot be implemented to reduce Medicaid spending for this class of drugs. Other policies, however, include preferred drug lists, prior authorization, copays, and generally tighter controls on high-cost drugs [26]. Some states, including California and Florida, both of which are among the top three states in terms of numbers of people with HIV/AIDS, are looking to increase substantially copays for certain groups of individuals on Medicaid. With the relatively high number of prescriptions people with HIV/AIDS need to fill, however, such a copay increase could constitute a significant barrier to obtaining all the drugs they need [26].

From 2001 through 2004, there were 5,660 new HIV/AIDS diagnoses reported to the Centers for Disease Control for persons 60 years old or older [3]. Moreover, one consequence of the expanded life span offered by antiretroviral treatment is that more elderly individuals will be coping with HIV/AIDS in the future. With the January 2006 implementation of a Medicare Part D prescription drug benefit, older people living with HIV/AIDS will be able to receive drug coverage from Medicare. Though drugs will be paid for differently under Medicare, Medicaid's experience with antiretrovirals during the last 15 years may shed some light on what will happen to Medicare spending over the next decade and a half, at least for this relatively small component of the overall Medicare budget.

## Conclusion

In this paper, we have documented the rise in spending on antiretrovirals by the U.S. Medicaid Program since 1991. We have shown that although most of the rise can be explained by rising utilization, some is due to rising prices of both newer medications and those already established in the market. Medicaid will struggle for many years to come to keep costs contained to the greatest extent possible, making sure that access to drugs is not compromised. While there may be some relief from generic medications, the rate of innovation remains strong in the antiretroviral drug class, and we predict that, at least for the near future, costs will keep rising.

## Competing interests

The author(s) declare that they have no competing interests.

## Authors' contributions

YJ, JJG, and CMLK designed the study. YJ, PK, and XL analyzed the data. All five authors drafted and approved this version of the manuscript.
